# High-quality draft genome sequence of *Thermobifida halotolerans* DSM 44931

**DOI:** 10.1128/mra.00509-25

**Published:** 2025-08-20

**Authors:** Colleen B. Ahern, I-Min Chen, Marcel Huntemann, Natalia Ivanova, Nikos Kyrpides, Supratim Mukherjee, Krishnaveni Palaniappan, Christa Pennacchio, T. B. K. Reddy, Stephan Ritter, Alexander Spunde, Dimitrios Stamatis, Peng Wang, Tanja Woyke, Yu Zhang, Michelle A. O'Malley

**Affiliations:** 1 Department of Chemical Engineering, University of California, Santa Barbara, California, USA; 2 U.S. Department of Energy Joint Genome Institute, Lawrence Berkeley National Laboratory, Berkeley, California, USA; 3 Joint BioEnergy Institute (JBEI) 124489, Emeryville, California, USA; 4 Department of Bioengineering, University of California 8786, Santa Barbara, California, USA; University of Southern California, Los Angeles, California, USA

**Keywords:** bacteria, genome, thermophile, actinomycete, biotechnology

## Abstract

Here, we report the genome sequence of *Thermobifida halotolerans* DSM 44931, a bacterium that was originally isolated from a salt mine in the Yunnan Province of China. This genome was sequenced using Pacific Biosciences sequencing technology and was assembled into 2 contigs in 2 scaffolds. It has a total length of 5,506,851 bp and a GC content of 71.16%. Functional annotation of this genome provides further metabolic insight into this species.

## ANNOUNCEMENT

*Thermobifida halotolerans* is an aerobic, gram-positive, and mesophilic bacterial species that was isolated from a salt mine in the Yunnan Province of China (1). It is a filamentous actinomycete whose genome encodes for enzymes that can degrade synthetic polymers and several types of biomass (2–5). It has previously been limited to a draft genome with 29× coverage, 74 scaffolds, and 371 contigs (GenBank accession number LIZN00000000) (6). This work presents an improved high-quality draft genome of *T. halotolerans* DSM 44931.

*T. halotolerans* DSM 44931 was purchased from the Leibniz Institute DSMZ and cultivated in 100 mL of Czapek peptone media for 7 days at 37°C. 20 mL of this culture was centrifuged at 4649 × g at room temperature for 8 minutes. High molecular weight genomic DNA was extracted from the pelleted sample using a modified cetyltrimethylammonium bromide protocol (7).

**Fig 1:**
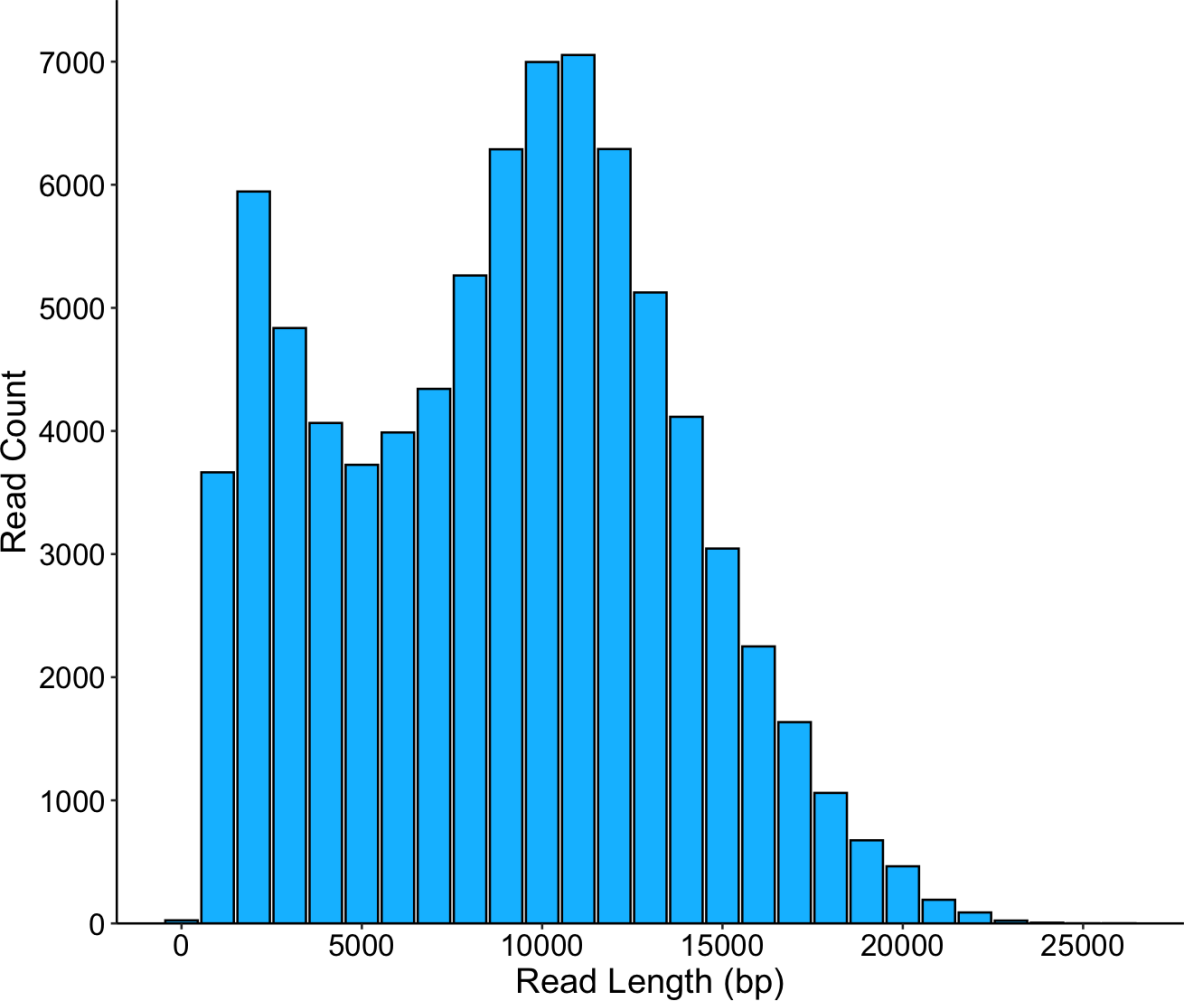
A histogram of the length frequencies of the CCS reads generated by the PacBio Sequel platform.

The quality and quantity of the extracted DNA were evaluated using an Agilent 2200 TapeStation and an Invitrogen Qubit 2.0 Fluorometer, respectively. The draft genome of *T. halotolerans* DSM 44931 was generated at the Department of Energy (DOE) Joint Genome Institute (JGI) using the Pacific Biosciences (PacBio) sequencing technology (8). Default parameters were used for all software unless otherwise specified. A >10 kb PacBio SMRTbellTM library was constructed and sequenced on the PacBio Sequel platform, which generated 81,163 high-fidelity CCS reads totaling 763,311,996 bp (Fig. 1). BBDuk in BBTools v38.98 was used to filter out low-quality reads (9). The input read coverage was 139.4×. Reads >5 kb were assembled with Flye v2.8.3 (10). The final draft assembly contained 2 contigs in 2 scaffolds, totaling 5,506,851 bp in size with a GC content of 71.16% (Table 1). The contigs were determined to be linear by Circlator v1.5.5 (11). The quality of this assembly was evaluated using tRNAscan-SE v2.0.4 (12) to count tRNAs; Barrnap v0.9-Dev (13) to determine the presence of the 5S, 16S, and 23S rRNA genes; and CheckM2 v1.1.0 (14) to estimate completeness and contamination (Table 1).

**TABLE 1:** Genome assembly statistics, quality assessment, and annotation statistics for *T. halotolerans* DSM 44931

Metric	Assembly statistic	
Total number of scaffolds	2	
Total number of contigs	2	
Total scaffold sequence length (bp)	5,506,851	
Total contig sequence length (bp)	5,506,851	
Scaffold N50 (bp)	5,450,929	
Contig N50 (bp)	5,450,929	
Pre-filtered CCS read N50 (bp)	11,849	
Largest contig (bp)	5,450,929	
Number of scaffolds >50 kb	2	
Percent of genome in scaffolds >50 kb	100%	
Percent of reads assembled	100%	

Metric	Assembly quality assessment	
5S rRNA gene presence	Yes	
16S rRNA gene presence	Yes	
23S rRNA gene presence	Yes	
tRNA count	54	
Estimated completeness	99.41%	
Estimated contamination	1.09%	

Genome annotation	Number	% of total
DNA, total number of bases	5,506,851	100.00%
DNA coding number of bases	4,707,236	85.48%
DNA G + C number of bases	3,918,505	71.16%^*a*^
DNA scaffolds	2	100.00%
CRISPR arrays	22	
Genes total number	5,051	100.00%
Protein-coding genes	4,961	98.22%
Regulatory and miscellaneous features	21	0.42%^*b*^
RNA genes	69	1.37%
rRNA genes	12	0.24%
5S rRNA	4	0.08%
16S rRNA	4	0.08%
23S rRNA	4	0.08%
tRNA genes	54	1.07%
Other RNA genes	3	0.06%
Protein-coding genes with function prediction	4,016	79.51%
without function prediction	945	18.71%
Protein-coding genes with enzymes	1,151	22.79%
Protein-coding genes connected to KEGG pathways	1,177	23.30%
not connected to KEGG pathways	3,784	74.92%
Protein-coding genes connected to KEGG Orthology (KO)	2,017	39.93%
not connected to KO	2,944	58.29%
Protein-coding genes connected to MetaCyc pathways	985	19.50%
not connected to MetaCyc pathways	3,976	78.72%
Protein-coding genes with COGs	3,404	67.39%
with Pfam	3,809	75.41%
with TIGRfam3	1,104	21.86%
with SMART	1,044	20.67%
with SUPERFam	3,979	78.78%
with CATH FunFam	2,973	58.86%
in internal clusters	1,317	26.07%
Protein-coding genes coding signal peptides	168	3.33%
Protein-coding genes coding transmembrane proteins	1,082	21.42%
COG clusters	1,609	34.74%
KOG clusters	0	0.00%
Pfam clusters	2,224	10.12%
TIGRfam clusters	893	19.90%

^*a*^ GC percentage shown as count of G's and C's divided by the total number of bases. The total number of bases is not necessarily synonymous with a total number of G's, C's, A's, and T's.

^*b*^ Regulatory or miscellaneous genes are genes that are not classified as a protein-coding gene, a type of RNA, or a pseudogene, but as 'unknown' or 'other' by the source provider.

The genome draft assembly was annotated using the JGI Integrated Microbial Genomes (15) annotation pipeline v5.1.9 (16). INFERNAL v1.1.2 (17) was used to search against the Rfam 13.0 (18) database (excluding tRNA and CRISPR models) to identify structural RNAs and regulatory motifs; GeneMark.hmm-2 v1.05 (19) and Prodigal v2.6.3 (20) to identify protein-coding genes; tRNAscan-SE v2.0.4 (12) to identify tRNAs; and CRT v1.8.2 (21) to predict CRISPR arrays. Functional annotation was assigned to the genes via lastal 1256 (22) and HMMER v3.1b2 (23) using the following databases: IMG-NR 20211118 (24), SMART 01_06_2016 (25), COG 2003 (26), TIGRFAM v15.0 (27), SuperFamily v1.75 (28), Pfam v34.0 (29), and Cath-Funfam v4.2.0 (30). Topological annotation of protein-coding genes was assigned using SignalP 4.1 (31) and decodeanhmm 1.1g (32). These annotations categorize this genome into protein-coding genes, regulatory and miscellaneous features, and RNA genes; furthermore, they reveal predicted metabolic roles and clustering of the protein-coding genes (Table 1).

Overall, this high-quality draft genome improves our understanding of *T. halotolerans* biology and our ability to use this species and other actinomycetes for biotechnological applications.

## Data Availability

The genome sequence was deposited to GenBank under the accession number JBGBYW000000000. The raw reads have been deposited in the NCBI SRA under the accession number SRP583730. Additional data can be explored or downloaded from the JGI Integrated Microbial Genomes with Microbiomes (IMG/M) portal using the NCBI BioProject accession number PRJNA1115251.
